# Lasing with Topological Weyl Semimetal

**DOI:** 10.1038/s41598-020-59423-3

**Published:** 2020-02-20

**Authors:** Güneş Oktay, Mustafa Sarısaman, Murat Tas

**Affiliations:** 1 Department of Physics, Istanbul University, 34134 Vezneciler Istanbul, Turkey; 2 Department of Physics, Gebze Technical University, 41400 Kocaeli, Turkey

**Keywords:** Optical physics, Topological matter, Lasers, LEDs and light sources, Quantum optics, Quantum mechanics

## Abstract

Lasing behavior of optically active planar topological Weyl semimetal (TWS) is investigated in view of the Kerr and Faraday rotations. Robust topological character of TWS is revealed by the presence of Weyl nodes and relevant surface conductivities. We focus our attention on the surfaces where no Fermi arcs are formed, and thus Maxwell equations contain topological terms. We explicitly demonstrate that two distinct lasing modes arise because of the presence of effective refractive indices which lead to the birefringence phenomena. Transfer matrix is constructed in such a way that reflection and transmission amplitudes involve 2 × 2 matrix-valued components describing the bimodal character of the TWS laser. We provide associated parameters of the topological laser system yielding the optimal impacts. We reveal that gain values corresponding to the lasing threshold display a quantized behavior, which occurs due to topological character of the system. Our proposal is supported by the corresponding graphical demonstrations. Our observations and predictions suggest a concrete way of forming TWS laser and coherent perfect absorber; and are awaited to be confirmed by an experimental realization based on our computations.

## Introduction

Introducing topological ideas into the material science has led to the emergence of a new and impressive field of physics, topological materials^1,2^. Especially, their interplay with electromagnetic waves offers remarkable applications in aspects of photonics, yielding a recent field of physics, topological photonics^3,4^. Recently, numerous active studies are witnessed in this field extending to various intriguing directions, which also help various aspects of non-Hermitian quantum mechanics to be understood in view of topological perspective. In this respect, recent study of constructing a theoretical modeling of topological insulator lasers and their accompanying experimental realization attracted attention to this field, which has served to alter current understanding of the relation between disorder and lasing, and has opened exciting possibilities at the interface of topological physics and laser science, such as topologically protected transport in systems with gain^5,6^. Motivated by these recent findings, we build up a theoretical modeling of a topological Weyl semimetal (TWS) laser by employing the transfer matrix approach in this paper. We reveal that topological protection of the surface states gives rise to bimodal topological laser which requires quantized gain values for the realization of the lasing threshold condition.

The TWS, one of leading candidates of topological materials, has attracted much attention in recent years for its novel gapless band structure in the bulk and exotic Fermi arcs on the surface states. Theoretical predictions of this exclusive phase of topological materials has been made since 2011^7–11^, and the first experimental verification of this phase was reported in TaAs material in 2015^12,13^. Recently, great interest in the TWS phase has arisen due to the possibility of realization of this phase in certain condensed matter systems. There are various predictions of solid materials as potential TWS candidates^14–16^, although their energy structures and topological properties are not perfectly consistent with an ideal TWS. In fact, an ideal TWS proposed in^14^ is rare in nature, and makes the analysis of the corresponding properties of TWS, i.e., spin texture of the boundary states, the flat bands, and the transport properties, quite difficult^17,18^. Their interaction with electromagnetic waves is thus quite intriguing, and gives rise to substantial applications of them, especially in developing new photonics devices. In this study, we fulfill a comprehensive treatment of electromagnetic scattering of these attractive materials just to unveil their lasing features and corresponding CPA applications.

Optical properties of TWS materials can be specified via their optical conductivities. For this purpose, the bulk and surface conductivity tensors taking into account all possible combinations of the optical transitions involving bulk and surface electron states can be employed. Thus, one can show how the information about electronic structure of TWS materials, such as the position and separation of Weyl nodes, Fermi energy, and Fermi arc surface states, can be extracted unambiguously from measurements of the dispersion, transmission, reflection, and polarization of electromagnetic waves^19–21^. As for the optical behaviors of TWS in a laser pulse, the femtosecond dynamics of electrons in such materials is highly irreversible, i.e., the residual electron population after the pulse is comparable to the conduction-band population during the pulse. Irreversibility of ultrafast electron dynamics is determined not only by the band gap of the material, but also by the profile and magnitude of the interband dipole elements. In the case of circularly-polarized pulse, response of the system to the linear probe pulse is highly dependent on the intrinsic chirality of the Weyl nodes. The magnitude of the charge transfer strongly depends on the direction of polarization^22–24^.

An optically active laser system can be obtained by means of the existence of spectral singularities^25–27^. Hence, we mount a homogeneous gain environment in a planar TWS slab such that TWS properties are preserved in a way that corresponding Weyl nodes are not affected, and Fermi arcs are present on its surfaces. Once electromagnetic waves are incident on a surface which does not contain a Fermi arc, polarization directions of the reflected and transmitted waves change due to the Kerr and Faraday rotations on the surfaces. This leads to the modified Maxwell equations involving topological terms in the source parts. Solution of these equations reveals coupled Helmholtz equations whose solutions yield a 4 × 4 transfer matrix. We show that the form of the transfer matrix provides a basis for the bimodal topological laser. This is characteristics of a TWS laser system such that purely outgoing waves cause rotated output intensity due to the Kerr and Faraday rotations. A coherent perfect absorber (CPA) using TWS can be thus obtained by adjusting appropriate polarizations given by the Faraday rotations together with exact phase and amplitude modulations^28–31^. This happens by virtue of the fact that CPA has the time reversal symmetry of a laser.

**Figure 1:**
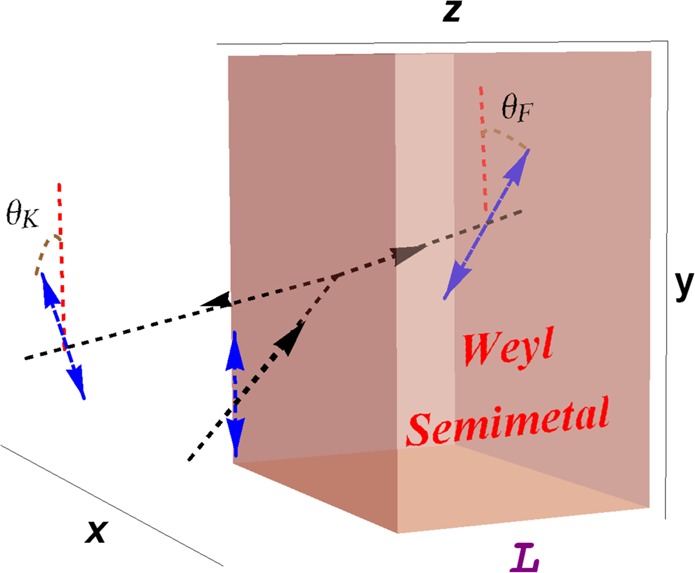
The TE mode configuration for the interaction of a electromagnetic wave with the Weyl semimetal slab. The wave is emitted on the slab by an angle *θ* which is measured from the normal to the surface, and direction of the polarization is rotated by an angle of *θ*_*F*_ and *θ*_*K*_ inside and outside of the slab respectively.

Although original problem is one dimensional, Kerr and Faraday rotations turn the problem into two-dimensional plane, see Fig. 1. Therefore, reflection and transmission amplitudes are expressed by means of 2 × 2 matrices. Dimension of these matrices decides bimodal structure of a TWS laser system. The spectral singularities for each mode are established by the divergent characteristics of reflection and transmission matrices. Hence, we obtain uni- or bimodal spectral singularity conditions with/without dispersion effects. We look for practical ways to improve the efficiency of the topological laser and CPA systems through the Supplemented Material features. It is noted that our formalism is satisfied by all TWS type materials.

We find complete solutions, schematically demonstrate their behaviors and deduce the effects of various parameter choices yielding laser conditions in the framework that we develop. We reveal the optimal control of parameters in a TWS uni- or bimodal slab laser, which include gain coefficient, incidence angle, slab thickness, and Weyl node separation. These optimal parameters give rise to a desired outcome of achieving rotated outgoing waves in the uni- or bimodal TWS laser system. We also present the way to find exact conditions causing the achievement of TWS CPA with equal amplitude and phase values of ingoing waves which are obtained by adjusting correct Faraday rotation angles. Our method, and thus results guide possible experimental studies in this direction for all proposed TWS slab materials of practical concern.

## TE Mode Solution and Transfer Matrix

**Figure 2:**
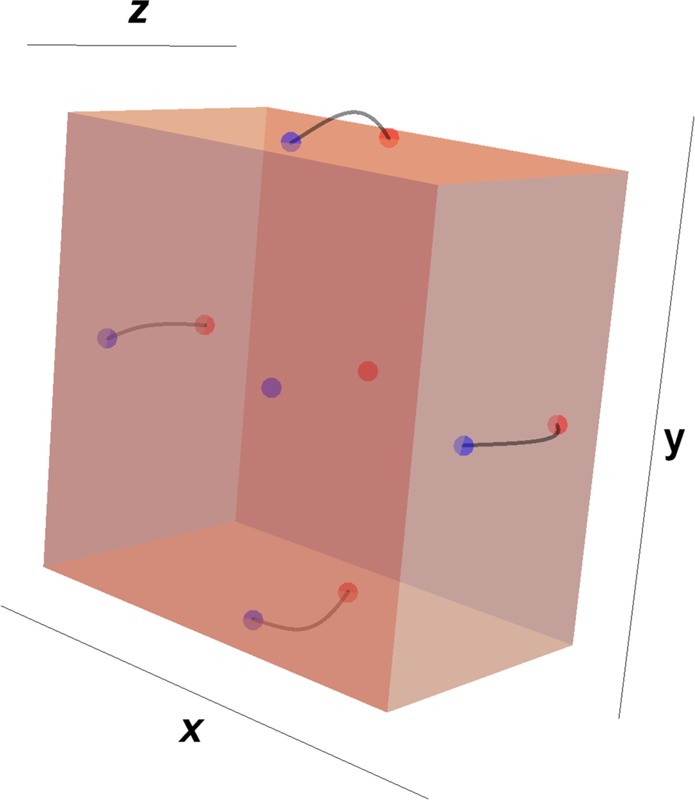
Schematic representation of locations of the Weyl nodes along the *k*_*z*_-axis in bulk Brillouin zone, which are the sources (green dot) and sinks (pink dot) of the Berry curvature. Fermi arcs corresponding to the Weyl nodes are formed on the relevant surfaces.

Consider a linear homogeneous and optically active gain slab system which is made up of a TWS material whose Weyl nodes are aligned along the *z*-axis as depicted in Fig. 1. The slab is designed in such a way that it has a thickness *L* and a complex refractive index ${\mathfrak{n}}$ which is uniform between the end-faces of the slab in the region 0 < *z* < *L*. Interaction of this TWS optical slab with the electromagnetic waves requires an elaborate analysis of the properties of TWS in view of topological and magnetoelectric optical effects. Topological features are crucial based on the alignment of the Weyl nodes which specify locations of the Fermi arcs on the faces of the slab. In our set-up, Fermi arcs appear on the side faces of the slab along the *z*-direction since the Weyl nodes are oriented in *z*-axis as shown in Fig. 2. In the light of this optical setup, Maxwell equations turn out to include topological terms (see Appendix A for the derivation of the topological terms) which are shown to take the form of

1 $$\overrightarrow{{\rm{\nabla }}}\cdot \overrightarrow{{\mathscr{D}}}=\rho (z)+\beta \overrightarrow{b}\cdot \overrightarrow{{\mathscr{B}}},\,\,\,\,\,\,\,\overrightarrow{{\rm{\nabla }}}\cdot \overrightarrow{{\mathscr{B}}}=0,$$

2 $$\overrightarrow{{\rm{\nabla }}}\times \overrightarrow{{\mathscr{H}}}-{{\rm{\partial }}}_{t}\overrightarrow{{\mathscr{D}}}=\overrightarrow{{\mathscr{J}}}(z)-\beta \overrightarrow{b}\times \overrightarrow{{\mathscr{E}}},\,\,\,\,{{\rm{\partial }}}_{t}\overrightarrow{{\mathscr{B}}}+\overrightarrow{{\rm{\nabla }}}\times \overrightarrow{{\mathscr{E}}}=\overrightarrow{0}.$$

Here $\beta :=\,2\alpha /\pi {Z}_{0}$ is a constant, $\alpha :=\,{e}^{2}/4\pi {\varepsilon }_{0}\hslash c$ is the fine structure constant, ${Z}_{0}:=\,\sqrt{{\mu }_{0}/{\varepsilon }_{0}}$ is the vacuum impedance, *e* is the charge of an electron, and $c:=1/\sqrt{{\varepsilon }_{0}{\mu }_{0}}$ is the speed of light in vacuum. The vector notation $\overrightarrow{{b}}$ specifies the distance between two Weyl nodes which are aligned in the *z*-direction and is given explicitly by $\overrightarrow{b}(z)=b(z){\hat{e}}_{z}$, and $b(z)=b{\mathfrak{u}}(z)\,{\mathfrak{u}}(L-z)$, where ${\mathfrak{u}}(z)$ is the Heaviside step function defined as

$${\mathfrak{u}}(z)\,:=\,\{\begin{array}{ll}0 & {\rm{for}}\,z < 0\\ 1 & {\rm{for}}\,z > 0\end{array}$$

Notice that $\overrightarrow{{\mathscr{E}}}$ and $\overrightarrow{{\mathscr{B}}}$ represent the electric and magnetic fields, respectively, and are expressed by $\overrightarrow{{\mathscr{D}}}$ and $\overrightarrow{{\mathscr{H}}}$ fields via the following constitutive relations

$$\overrightarrow{{\mathscr{D}}}\,:=\,\tilde{\varepsilon }\overrightarrow{{\mathscr{E}}},\,\overrightarrow{{\mathscr{B}}}\,:=\,\tilde{\mu }\overrightarrow{{\mathscr{H}}},$$

where $\tilde{\varepsilon }$ and $\tilde{\mu }$ are, respectively, permittivity and permeability of the relevant environment in which the electromagnetic wave propagates. These are described by $\tilde{\varepsilon }\,:=\,{\varepsilon }_{0}\varepsilon$ and $\tilde{\mu }\,:=\,{\mu }_{0}\mu$, respectively, in terms of the vacuum permittivity and permeability, where we defined

3 $$\varepsilon (z)\,:=\,\{\begin{array}{ll}{\varepsilon }_{b}+\frac{i{\sigma }_{yy}}{{\varepsilon }_{0}\omega } & {\rm{for}}\,z\in [0,L],\\ 1 & {\rm{otherwise}}\end{array}$$

4 $$\mu (z)\,:=\,\{\begin{array}{ll}1+{\chi }_{m} & {\rm{for}}\,z\in [0,L],\\ 1 & {\rm{otherwise}}\end{array}$$

*ε*_*b*_ is the bound charge contribution, *χ*_*m*_ is the magnetic susceptibility of the TWS provided that it exhibits a magnetic characteristics. In this study, we assume that TWS is endowed with a rather weak magnetism such that we may ignore it (For the sake of full discussion, we keep its presence till the end.). Notice that ${{\mathfrak{n}}}^{2}\,:=\,\varepsilon \mu$ within the slab according to the expressions (3) and (4), where ${\mathfrak{n}}$ corresponds to the complex-valued refractive index of TWS, and is described by real and imaginary parts as follows

5 $${\mathfrak{n}}\,:=\,\eta +i\kappa $$

We observe that free charges and currents in our optical setup appear only on surfaces of the TWS slab on which the incident wave emerges and no Fermi arc is present. This is because of the topological character of TWS which leads to form conductivities only on the surfaces, whereas it behaves as a semimetal inside the TWS medium. Therefore, surface charge density *ρ*^*s*^(*z*) and conductivity *σ*^*s*^(*z*) can be written as

$${\rho }^{s}(z)\,:=\,{\rho }^{(1)}\delta (z)+{\rho }^{(2)}\delta (z-L),$$

$${\sigma }^{s}(z)\,:=\,{\sigma }^{(1)}\delta (z)+{\sigma }^{(2)}\delta (z-L),$$

where *ρ*^(*j*)^ and *σ*^(*j*)^ are, respectively, the free charge and conductivity on the *j*-th layer, with *j* = 1, 2. Notice that *ρ*^*s*^(*z*) and *σ*^*s*^(*z*) are associated to each other by the continuity equation

6 $$\overrightarrow{\nabla }\cdot \overrightarrow{{{\mathscr{J}}}^{s}}+{\partial }_{t}{\rho }^{s}(z)=0$$

for the electric current density given by $\overrightarrow{{{\mathscr{J}}}^{s}}\,:=\,{\sigma }^{s}(z)\overrightarrow{{\mathscr{E}}}$. Surface conductivity *σ*^*s*^ has a tensorial expression, and thus we can demonstrate the surface current $\overrightarrow{{{\mathscr{J}}}^{s}}$ as $\overrightarrow{{{\mathscr{J}}}_{\alpha }^{s}}={\sigma }_{\alpha \beta }^{s}{\overrightarrow{E}}_{\beta }$. In our case,

7 $${\sigma }_{yy}^{s}=\frac{{e}^{2}{k}_{c}}{3\pi h{\hat{\omega }}_{c}}\{1-i[{\hat{\omega }}_{c}^{2}+\,\mathrm{ln}|1-{\hat{\omega }}_{c}^{2}|]\}$$

8 $${\sigma }_{yx}^{s}=\frac{{e}^{2}b}{\pi h}+\frac{\alpha c}{3\pi {v}_{F}}\,\mathrm{ln}|1-{\hat{\omega }}_{c}^{2}|$$

where ${\hat{\omega }}_{c}\,:=\,2{\omega }_{c}/\omega$, ${\omega }_{c}\,:=\,{v}_{F}{k}_{c}$, *v*_*F*_ is the Fermi velocity, *k*_*c*_ is the momentum cut-off and *k* ≤ *k*_*c*_, see Appendix for the details of the derivations of (7) and (8). We emphasize that the conductivity ${\sigma }_{yx}^{s}$ is responsible for the Kerr and Faraday rotations inside and outside the TWS. Consider time harmonic electromagnetic waves having TE mode solutions corresponding to our geometrical design such that the wave can be regarded as obliquely incident in the form, see Fig. 1 (Time harmonic electric field corresponds to $\overrightarrow{{\mathscr{E}}}(\overrightarrow{r},t)\,:=\,\overrightarrow{E}(\overrightarrow{r})\,{e}^{-i\omega t}$, similarly for $\overrightarrow{{\mathscr{B}}}(\overrightarrow{r},t)$, $\overrightarrow{{\mathscr{D}}}(\overrightarrow{r},t)$ and $\overrightarrow{{\mathscr{H}}}(\overrightarrow{r},t)$ fields.)

9 $$\overrightarrow{E}(\overrightarrow{r})={\mathscr{E}}(z){e}^{i{k}_{x}x}{\hat{e}}_{y},$$

In this notation, ${\hat{e}}_{j}$ denotes the unit vector along the *j*-axis, with *j* = *x*, *y* and *z*, and *k*_*j*_ is the component of wavevector $\overrightarrow{k}$ in the *j*-direction, where $\overrightarrow{k}$ is given by

$$\overrightarrow{k}={k}_{x}{\hat{e}}_{x}+{k}_{z}{\hat{e}}_{z}$$

such that *k*_*x*_ = *k* sin*θ*, *k*_*z*_ = *k* cos*θ*, and *θ* ∈ [−90°, 90°] represents the incident angle. We notice that polarization direction of the emitted wave will be twisted once it is reflected and refracted at the interface of TWS. Reflected waves give rise to the Kerr rotation while the refracted waves to the Faraday rotation within the slab, see Fig. 1.

The Maxwell equations in (1) and (2) can be manipulated to give 3-dimensional Helmholtz equation associated with the TE mode states, and corresponding magnetic field $\overrightarrow{H}$ as follows

10 $$[{\nabla }^{2}+{k}^{2}\varepsilon (z)\mu (z)]\overrightarrow{E}-i\beta k{Z}_{0}\overrightarrow{b}\times \overrightarrow{E}=0,$$

11 $$\overrightarrow{H}=-\,\frac{i}{k{Z}_{0}\mu (z)}\overrightarrow{\nabla }\times \overrightarrow{E},$$

TE mode solution of (10) in view of the Kerr and Faraday rotations can be established by means of the following one-dimensional coupled Helmholtz equations (We obtain the TE mode solution of the Kerr and Faraday rotated optical system by specifying $\overrightarrow{E}={E}_{x}{\hat{e}}_{x}+{E}_{y}{\hat{e}}_{y}$, where ${E}_{x}(\overrightarrow{r})={{\mathscr{E}}}_{x}(z){e}^{i{k}_{x}x}$ and ${E}_{y}(\overrightarrow{r})={{\mathscr{E}}}_{y}(z){e}^{i{k}_{x}x}$.)

12 $${{\mathscr{E}}{\prime\prime} }_{x}+{k}_{z}^{2}{\mathfrak{z}}(z){{\mathscr{E}}}_{x}+i\beta k{Z}_{0}b\,{{\mathscr{E}}}_{y}=0$$

13 $${{\mathscr{E}}{\prime\prime} }_{y}+{k}_{z}^{2}{\mathfrak{z}}(z){{\mathscr{E}}}_{y}-i\beta k{Z}_{0}b\,{{\mathscr{E}}}_{x}=0$$

where a prime denotes derivative with respect to *z*. The piecewise constant function ${\mathfrak{z}}(z)$ is specified by

$${\mathfrak{z}}(z)\,:=\,\{\begin{array}{cc}{\tilde{{\mathfrak{n}}}}^{2} & {\rm{for}}\,z\in [0,L],\\ 1 & {\rm{otherwise}}\end{array}$$

$$\tilde{{\mathfrak{n}}}\,:=\,\sec \,\theta \sqrt{{{\mathfrak{n}}}^{2}-{\sin }^{2}\theta }$$

We next introduce the following scaled variables for the convenience of subsequent expressions

14 $${\bf{x}}\,:=\,\frac{x}{L},\,{\bf{z}}\,:=\,\frac{z}{L},\,{\mathfrak{K}}\,:=\,L{k}_{z}=kL\,\cos \,\theta .$$

Solutions of (12) and (13) are attained once we split them in uncoupled modes as follows

$${\psi }_{\pm }({\bf{z}})\,:=\,{{\mathscr{E}}}_{x}(L{\bf{z}})\pm i{{\mathscr{E}}}_{y}(L{\bf{z}})$$

where *ψ*_±_ are the solutions of Schrödinger equations

15 $$-{\psi {\prime\prime} }_{\pm }+{v}_{\pm }({\bf{z}}){\psi }_{\pm }={{\mathfrak{K}}}^{2}{\psi }_{\pm }$$

for the potentials given by ${v}_{\pm }({\bf{z}})={{\mathfrak{K}}}^{2}{{\rm{z}}}_{\pm }({\bf{z}})$. Here ${{\mathfrak{z}}}_{\pm }({\bf{z}})$ is defined as

16 $${{\mathfrak{z}}}_{\pm }({\bf{z}})\,:=\,{\mathfrak{z}}({\bf{z}})\pm \frac{2\alpha Lb({\bf{z}})}{\pi {\mathfrak{K}}\,\cos \,\theta }$$

Notice that refractive indices ${\tilde{{\mathfrak{n}}}}_{\pm }=\sqrt{{\tilde{{\mathfrak{n}}}}^{2}\pm 2\alpha bL/\pi {\mathfrak{K}}\,\cos \,\theta }$ within the TWS slab lead to the birefrigence effect because of the presence of uncoupled modes in (16). In view of (11), (15) and (16), one finds components of the electric field $\overrightarrow{E}$ and magnetic field $\overrightarrow{H}$ as in Table 1, where the quantities ${{\mathscr{F}}}_{\pm }$ and ${{\mathscr{G}}}_{\pm }$ are defined in different regions of optical TWS slab system by

$$\begin{array}{cc}{{\mathscr{F}}}_{\pm }\,:=\,\{\begin{array}{ll}{A}_{1}^{(-)}{e}^{i{\mathfrak{K}}{\bf{z}}}\pm {C}_{1}^{(-)}{e}^{-i{\mathfrak{K}}{\bf{z}}} & {\rm{for}}\,{\bf{z}} < 0,\\ {B}_{1}^{(+)}{e}^{i{{\mathfrak{K}}}_{+}{\bf{z}}}\pm {B}_{2}^{(+)}{e}^{-i{{\mathfrak{K}}}_{+}{\bf{z}}} & {\rm{for}}\,0 < {\bf{z}} < 1,\\ {A}_{1}^{(+)}{e}^{i{\mathfrak{K}}{\bf{z}}}\pm {C}_{1}^{(+)}{e}^{-i{\mathfrak{K}}{\bf{z}}} & {\rm{for}}\,{\rm{z}} > 1.\end{array} & {{\mathscr{G}}}_{\pm }\,:=\,\{\begin{array}{ll}{A}_{2}^{(-)}{e}^{i{\mathfrak{K}}{\bf{z}}}\pm {C}_{2}^{(-)}{e}^{-i{\mathfrak{K}}{\bf{z}}} & {\rm{for}}\,{\bf{z}} < 0,\\ {B}_{1}^{(-)}{e}^{i{{\mathfrak{K}}}_{-}{\bf{z}}}\pm {B}_{2}^{(-)}{e}^{-i{{\mathfrak{K}}}_{-}{\bf{z}}} & {\rm{for}}\,0 < {\bf{z}} < 1,\\ {A}_{2}^{(+)}{e}^{i{\mathfrak{K}}{\bf{z}}}\pm {C}_{2}^{(+)}{e}^{-i{\mathfrak{K}}{\bf{z}}} & {\rm{for}}\,{\rm{z}} > 1.\end{array}\end{array}$$

**Table 1:** Components of $\overrightarrow{E}$ and $\overrightarrow{H}$ fields existing inside and outside the TWS slab.

Components of $\overrightarrow{E}$-field	Components of $\overrightarrow{H}$-field
${E}_{x}=\frac{({{\mathscr{F}}}_{+}+{{\mathscr{G}}}_{+})}{2}\,{e}^{i{\mathfrak{K}}{\bf{x}}\tan \theta }$	${H}_{x}=\frac{i\,\cos \,\theta }{2{Z}_{0}}[\sqrt{{{\rm{z}}}_{+}}{{\mathscr{F}}}_{-}-\sqrt{{{\rm{z}}}_{-}}{{\mathscr{G}}}_{-}]{e}^{i{\mathfrak{K}}{\bf{x}}\tan \theta }$
${E}_{y}=\frac{-i({{\mathscr{F}}}_{+}-{{\mathscr{G}}}_{+})}{2}\,{e}^{i{\mathfrak{K}}{\bf{x}}\tan \theta }$	${H}_{y}=\frac{\cos \,\theta }{2{Z}_{0}\mu }[\sqrt{{{\rm{z}}}_{+}}{{\mathscr{F}}}_{-}+\sqrt{{{\rm{z}}}_{-}}{{\mathscr{G}}}_{-}]{e}^{i{\mathfrak{K}}{\bf{x}}\tan \theta }$
*E*_*z*_ = 0	${H}_{z}=-\,\frac{i\,\sin \,\theta }{2{Z}_{0}}[{{\mathscr{F}}}_{+}-{{\mathscr{G}}}_{+}]{e}^{i{\mathfrak{K}}{\bf{x}}\tan \theta }$

Here we introduced the quantity ${{\mathfrak{K}}}_{j}$ as follows

17 $${{\mathfrak{K}}}_{j}\,:=\,{\mathfrak{K}}{\tilde{{\mathfrak{n}}}}_{j}.$$

The complex coefficients ${A}_{j}^{(\pm )},{B}_{j}^{(\pm )}$ and ${C}_{j}^{(\pm )}$ are possibly ${\mathfrak{K}}$-dependent, and related to each other by means of standard boundary conditions. Appropriate boundary conditions in our configuration of the optical system are described by the fact that tangential components of $\overrightarrow{E}$-fields and normal components of $\overrightarrow{B}$-fields are continuous across the interfaces while tangential components of $\overrightarrow{H}$-fields are discontinuous by an amount equal to the surface current density $\overrightarrow{{\mathscr{J}}}$ which is expressed by the conductivities on the corresponding surfaces. See Appendix for the associated boundary conditions. Hence, the transfer matrix following from the boundary conditions are obtained as follows

$$(\begin{array}{c}{{\bf{A}}}^{(+)}\\ {{\bf{C}}}^{(+)}\end{array})={\mathbb{M}}({\mathfrak{K}})(\begin{array}{c}{{\bf{A}}}^{(-)}\\ {{\bf{C}}}^{(-)}\end{array}).$$

Here **A**^(±)^ and **C**^(±)^ are the column matrices which represent the coefficients of right and left moving waves outside the TWS slab, and are given by

$${{\bf{A}}}^{(\pm )}=(\begin{array}{c}{A}_{1}^{(\pm )}\\ {A}_{2}^{(\pm )}\end{array}),\,{{\bf{C}}}^{(\pm )}=(\begin{array}{c}{C}_{1}^{(\pm )}\\ {C}_{2}^{(\pm )}\end{array}),$$

and ${\mathbb{M}}({\mathfrak{K}})$ is the 4 × 4 transfer matrix^32^ which is expressed by

18 $${\mathbb{M}}({\mathfrak{K}})=(\begin{array}{cc}{{\bf{T}}}^{l}-{{\bf{R}}}^{l}{{\bf{R}}}^{r}{{\bf{T}}}^{-r} & {{\bf{R}}}^{r}{{\bf{T}}}^{-r}\\ -{{\bf{R}}}^{l}{{\bf{T}}}^{-r} & {{\bf{T}}}^{-r}\end{array})$$

Once we impose the reciprocity principle, we understand that **T**^*l*^ = **T**^*r*^ = **T**. Notice that the transfer matrix (18) gives rise to deduce all information about the lasing properties of TWS optical configuration since it contains valuable information about reflection and transmission amplitudes. Lasing threshold condition is then given by the spectral singularity expression which is given by the requirement ${{\mathbb{M}}}_{22}=0$ corresponding to the real ${\mathfrak{K}}$ values. Thus, transmission and right/left reflection amplitudes are divergent at spectral singularity points. In our TWS configuration, ${{\mathbb{M}}}_{22}$ is obtained as follows

19 $${{\mathbb{M}}}_{22}={{\bf{T}}}^{-1}=-(\begin{array}{cc}{\zeta }_{-}({\nu }_{+};{\nu }_{-}) & {\gamma }_{-}({\nu }_{+};{\nu }_{-})\\ {\gamma }_{-}({\nu }_{-};{\nu }_{+}) & {\zeta }_{-}({\nu }_{-};{\nu }_{+})\end{array}){e}^{i{\mathfrak{K}}},$$

where ${\nu }_{j}\,:=\,({\sigma }_{j},{\tilde{{\mathfrak{n}}}}_{j})$, and ${\zeta }_{j}$, ${\gamma }_{j}:{\mathbb{C}}\to {\mathbb{C}}$ are continuous functions and specified by

$$\begin{array}{ccc}{\zeta }_{j}({\nu }_{+};{\nu }_{-}) & := & \frac{1}{2}\{({\sigma }_{+}-{\sigma }_{-}+2j)\cos \,{{\mathfrak{K}}}_{+}-\frac{i\mu {\sigma }_{+}^{2}}{{\tilde{{\mathfrak{n}}}}_{-}}\,\sin \,{{\mathfrak{K}}}_{-}+\,\frac{i[{\tilde{{\mathfrak{n}}}}_{+}^{2}-{\mu }^{2}({\sigma }_{+}+j)({\sigma }_{-}-j)]}{\mu {\tilde{{\mathfrak{n}}}}_{+}}\,\sin \,{{\mathfrak{K}}}_{+}\},\\ {\gamma }_{j}({\nu }_{+};{\nu }_{-}) & := & \frac{1}{2}\{{\sigma }_{+}\,\cos \,{{\mathfrak{K}}}_{-}+\frac{i\mu {\sigma }_{+}({\sigma }_{-}-1)}{{\tilde{{\mathfrak{n}}}}_{-}}\,\sin \,{{\mathfrak{K}}}_{-}-{\sigma }_{-}\,\cos \,{{\mathfrak{K}}}_{+}+\frac{i\mu {\sigma }_{-}({\sigma }_{-}-j)}{{\tilde{{\mathfrak{n}}}}_{+}}\,\sin \,{{\mathfrak{K}}}_{+}\},\end{array}$$

with the appropriate identification of *σ*_*j*_

20 $${\sigma }_{j}\,:=\,\frac{{\mu }_{0}\omega L}{2{\mathfrak{K}}}({\sigma }_{yy}+ij{\sigma }_{yx})=\frac{{Z}_{0}}{2\cos \,\theta }({\sigma }_{yy}+ij{\sigma }_{yx})$$

where *j* = + or − is implied. Thus, lasing threshold condition of TWS is obtained by means of the spectral singularities which are found by the real values of ${\mathfrak{K}}$ such that matrix components of ${{\mathbb{M}}}_{22}$ in Eq. 19 are set to zero. In view of this fact, we realize that each of the plus (“+”) and minus (“−”) modes corresponding to the plus/minus refractive indices may lead to form distinctive lasing conditions. This two-mode lasing arises due to the coupling behavior of solutions appearing in the TWS configurations, which is seen by virtue of the Kerr and Faraday rotations. Thus, plus/minus mode lasing is attained by the condition

21 $${\zeta }_{-}({\nu }_{\pm };{\nu }_{\mp })={\gamma }_{-}({\nu }_{\pm };{\nu }_{\mp })=0.$$

**Figure 3:**
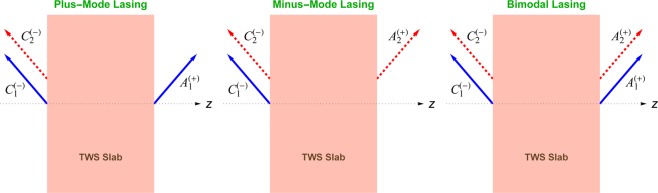
Configurations of uni- and bimodal lasing. Blue solid arrows correspond to lasing due to ${\tilde{{\mathfrak{n}}}}_{+}$, and red dashed arrows correspond to lasing due to ${\tilde{{\mathfrak{n}}}}_{-}$.

This in turn implies the conditions ${A}_{1}^{(-)}={A}_{2}^{(-)}={C}_{1}^{(+)}=0$ for the plus-mode and ${A}_{1}^{(-)}={A}_{2}^{(-)}={C}_{2}^{(+)}=0$ for the minus-mode lasing to generate purely outgoing waves. Bimodal-lasing is provided once all four conditions in (21) are imposed. Notice that plus-mode lasing yields lasing in both sides of the slab due to the effective refractive index ${\tilde{{\mathfrak{n}}}}_{+}$ and a lasing from left-hand side due to ${\tilde{{\rm{n}}}}_{-}$. Likewise, minus-mode lasing leads to a bidirectional lasing from ${\tilde{{\rm{n}}}}_{-}$ and a left side lasing due to ${\tilde{{\mathfrak{n}}}}_{+}$. This reveals the distinctive character of TWS laser slab. Figure 3 clearly demonstrates these lasing behaviors of plus and minus-modes.

## Lasing Conditions in Plus/Minus-Modes and Related Parameters

Uni- or bimodal lasing behavior of TWS slab described by Eq. 21 can be further analyzed to reveal conditions for the necessary parameters of the corresponding optical system. An immediate and simultaneous computation of two equations in (21) gives rise to the following expression for each individual mode

22 $${e}^{2i{{\mathfrak{K}}}_{j}}=-\,{{\mathscr{U}}}_{j}+\sqrt{{{\mathscr{U}}}_{j}^{2}+1}$$

with the following identifications

$${{\mathscr{U}}}_{j}\,:=\,\frac{{{\mathscr{X}}}_{j}{{\mathscr{Z}}}_{j}\pm {{\mathscr{Y}}}_{j}\sqrt{{{\mathscr{X}}}_{j}^{2}-{{\mathscr{Y}}}_{j}^{2}+{{\mathscr{Z}}}_{j}^{2}}}{{{\mathscr{Y}}}_{j}^{2}-{{\mathscr{Z}}}_{j}^{2}}$$

$${{\mathscr{X}}}_{j}\,:=\,\frac{1}{2}\{{{\mathscr{A}}}_{2j}^{2}-{{\mathscr{A}}}_{1j}^{2}+{\sigma }_{j}^{2}[{{\mathscr{A}}}_{3j}^{2}-{{\mathscr{A}}}_{4j}^{2}-{{\mathscr{A}}}_{5j}^{2}]\}$$

$${{\mathscr{Y}}}_{j}\,:=\,\frac{1}{2}\{{{\mathscr{A}}}_{2j}^{2}+{{\mathscr{A}}}_{1j}^{2}-{\sigma }_{j}^{2}[{{\mathscr{A}}}_{3j}^{2}+{{\mathscr{A}}}_{4j}^{2}]\}$$

$${{\mathscr{Z}}}_{j}\,:=\,{{\mathscr{A}}}_{1j}{{\mathscr{A}}}_{2j}+{\sigma }_{j}^{2}{{\mathscr{A}}}_{3j}{{\mathscr{A}}}_{4j}$$

$${{\mathscr{A}}}_{1j}\,:=\,{\sigma }_{j}-{\sigma }_{-j}-2,\,{{\mathscr{A}}}_{2j}\,:=\,\frac{{\tilde{{\mathfrak{n}}}}_{j}^{2}-{\mu }^{2}({\sigma }_{j}-1)({\sigma }_{-j}+1)}{\mu {\tilde{{\mathfrak{n}}}}_{j}}$$

$${{\mathscr{A}}}_{3j}\,:=\,\frac{\mu }{{\tilde{{\mathfrak{n}}}}_{-j}}[({\sigma }_{-j}-1)({\sigma }_{+}-{\sigma }_{-}+1-2j)+1]$$

$${{\mathscr{A}}}_{4j}\,:=\,\frac{{\mu }^{2}({\sigma }_{-j}+1)[({\sigma }_{-j}-1){{\mathfrak{a}}}_{j}+1]-j({\sigma }_{-j}-1){\tilde{{\mathfrak{n}}}}_{j}^{2}}{{\tilde{{\mathfrak{n}}}}_{-}{\tilde{{\mathfrak{n}}}}_{+}},$$

$${{\mathscr{A}}}_{5j}\,:=\,\frac{\sqrt{2}\mu }{{\tilde{{\mathfrak{n}}}}_{-j}},\,{{\mathfrak{a}}}_{j}\,:=\,1+j({\sigma }_{j}-1),$$

where the subindex *j* = + and − corresponds to the plus and minus modes. Uni- or bimodal lasing behavior of our TWS system is controlled by the expression (22). We realize that surface conductivities *σ*_*j*_ associated with each mode are the manifestation of bimodal lasing attitude, which stems from the presence of Weyl nodes, and thus the Kerr and Faraday rotations. If the conditions of forming TWS are removed or disturbed considerably, the condition of uni- or bimodal lasing in Eq. 22 is violated. Notice that (22) is a complex expression displaying the behavior of system parameters of our optical setup. The most appropriate system parameters should be chosen for the emergence of optimal impacts. Thus, it can be explored extensively by means of relevant physical quantities containing significant consequences by splitting into the real and imaginary parts. In this direction, refractive index of TWS described by Eq. 5 leads to the effective refractive indices which are bifurcated as follows

23 $${\tilde{{\mathfrak{n}}}}_{j}\approx {\tilde{\eta }}_{j}+i{\tilde{\kappa }}_{j},$$

with the identifications

$${\tilde{\eta }}_{j}\,:=\,\,\sec \theta \sqrt{{\eta }^{2}-{\sin }^{2}\theta +\frac{2j\alpha bL\,\cos \,\theta }{\pi {\mathfrak{K}}}},\,{\tilde{\kappa }}_{j}\,:=\,\frac{\eta \kappa {\sec }^{2}\theta }{{\tilde{\eta }}_{j}}.$$

Notice that expression for ${\tilde{{\mathfrak{n}}}}_{j}$ in Eq. 23 makes use of the condition that most of the materials including TWS satisfy

$$|\kappa |\ll \eta -1 < \eta .$$

Next we introduce the gain coefficient *g* by

24 $$g\,:=\,-\,2k\kappa =-\,\frac{4\pi \kappa }{\lambda }=-\,\frac{2{\mathfrak{K}}\kappa }{L\,\cos \,\theta }.$$

Therefore, uni- or bimodal spectral singularity condition expressed by Eq. 22 gives rise to the following expressions for the gain coefficient *g*_*j*_ and effective wavenumber ${{\mathfrak{K}}}^{j}$ (Here we use superindex *j* to avoid abuse of notation in Eq. 17. ${{\rm{K}}}^{j}$ simply refers to the wavenumber corresponding to the mode *j*).

25 $${g}_{j}=\frac{{\tilde{\eta }}_{j}\,\cos \,\theta }{2\eta L}\,\mathrm{ln}\,|{{\mathscr{V}}}_{j}|,$$

26 $$\,{{\mathfrak{K}}}^{j}=\frac{1}{2{\tilde{\eta }}_{j}}{\sin }^{-1}\{\frac{-{\rm{Im}}[{{\mathscr{U}}}_{j}]\pm \sqrt{{{\mathscr{W}}}_{j}}\,\sin (\frac{{\Phi }_{j}}{2})}{\sqrt{{{\mathscr{V}}}_{j}}}\},$$

where we define the associated quantities

$$\begin{array}{ccc}{{\mathscr{V}}}_{j} & := & {{\mathscr{W}}}_{j}+{\rm{Re}}{[{{\mathscr{U}}}_{j}]}^{2}+{\rm{Im}}{[{{\mathscr{U}}}_{j}]}^{2}\mp 2\sqrt{{{\mathscr{W}}}_{j}}[\sin (\frac{{\Phi }_{j}}{2}){\rm{Im}}[{{\mathscr{U}}}_{j}]+\,\cos (\frac{{\Phi }_{j}}{2}){\rm{Re}}[{{\mathscr{U}}}_{j}]],\\ {{\mathscr{W}}}_{j} & := & \sqrt{{\rm{Re}}{[{{\mathscr{U}}}_{j}]}^{4}+{({\rm{Im}}{[{{\mathscr{U}}}_{j}]}^{2}-1)}^{2}+2{\rm{Re}}{[{{\mathscr{U}}}_{j}]}^{2}({\rm{Im}}{[{{\mathscr{U}}}_{j}]}^{2}+1)},\\ {\Phi }_{j} & := & {\tan }^{-1}\{\frac{2{\rm{Re}}[{{\mathscr{U}}}_{j}]{\rm{Im}}[{{\mathscr{U}}}_{j}]}{1+{\rm{Re}}{[{{\mathscr{U}}}_{j}]}^{2}-{\rm{Im}}{[{{\mathscr{U}}}_{j}]}^{2}}\},\end{array}$$

and Re and Im correspond to the real and imaginary part of the relevant quantity, respectively. We explore the most appropriate system parameters through the expressions of gain coefficient *g*_*j*_ and ${{\mathfrak{K}}}^{j}$ in Eqs. 25 and 26 for the emergence of optimal impacts. Thus, a comprehensive analysis of the involvement of system parameters is required to observe the final outcome. For this purpose we exhibit the general behaviors of system parameters through the physically applicable gain coefficient g plots. But, notice that *g*_−_-value in (25) does not allow lasing for some wavelength configurations due to the presence of the parameter ${\tilde{\eta }}_{-}$. Although it is not explicitly seen in the expression for *g*_+_, the same restriction holds for *g*_+_ as well. This in turn implies that the distance *b* between the Weyl nodes in the Brillouin zone must satisfy

27 $$b < \frac{{\pi }^{2}({\eta }^{2}-{\sin }^{2}\theta )}{\alpha \lambda }.$$

In position space, this corresponds to

28 $$b{\prime}  > \frac{2\alpha \lambda }{\pi ({\eta }^{2}-{\sin }^{2}\theta )},$$

if we introduce $b\,:\,=2\pi /b{\prime}$. In (28), the lower bound of *b*′ can be identified by ${b{\prime} }_{c}\,:\,=2\alpha \lambda /\pi ({\eta }^{2}-{\sin }^{2}\theta )$. Thus we realize that the lower bound of *b*′ depends on the incidence angle *θ* and wavelength *λ*. But *θ* has little effect compared to *λ*. Therefore, once the wavelength is increased, corresponding ${b{\prime} }_{c}$ value is reduced in order to observe a lasing in both plus and minus modes. The following graphs display the justification of our calculations which are based on the following specifications of a nonmagnetic (*μ* ≈ 1) TaAs TWS slab system^33–37^

29 $$\begin{array}{c}\eta \approx 6,\,\lambda =1400\,{\rm{nm}},\,L=1\,{\rm{cm}},\\ \theta ={30}^{\circ }\,{\rm{and}}\,b{\prime} =0.05.\end{array}$$

**Figure 4:**
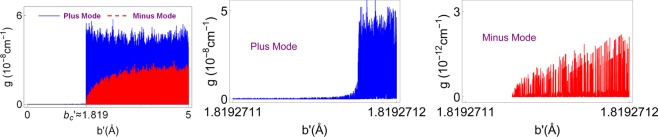
Gain coefficient as a function of Weyl node separation *b*′ corresponding to the plus and minus modes for the configuration of the TWS slab system given in (29). Here, ${b{\prime} }_{c}$ denotes the critical *b*′ value which corresponds to the minimum *b*′-value that allows lasing. Last two Figs. indicate the region that ${b{\prime} }_{c}$ takes effect. Horizontal gray line matches up to the zero gain limit.

**Figure 5:**
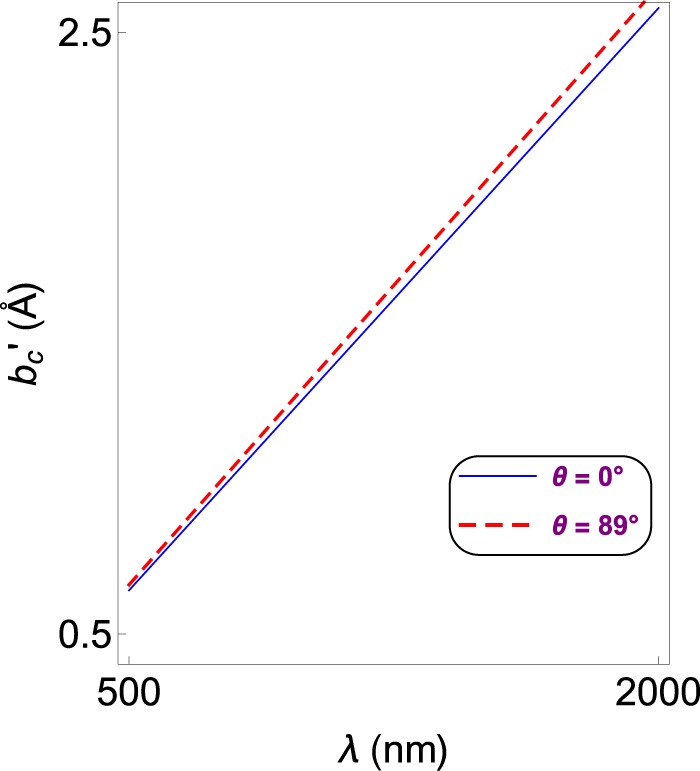
Dependence of critical ${b{\prime} }_{c}$-value on the wavelength *λ* for various incidence angles, corresponding to both plus and minus-mode lasing.

Figure 4 reveals the effect of the Weyl node separation *b*′ in position space on the gain coefficient *g* for the realization of lasing. Gain values are tightly packed. Notice that both plus and minus nodes give rise to a minimal bound ${b{\prime} }_{c}$, below which no lasing occurs. The best lasing impact with high quality factor is acquired by opting a TWS material whose Weyl node separation is around the critical *b*′-value. ${b{\prime} }_{c}$-value raises slightly by increasing the incidence angle *θ*, and significantly by the wavelength rise. Figure 5 shows how ${b{\prime} }_{c}$ varies with respect to wavelength *λ* and incidence angle *θ*.

**Figure 6:**
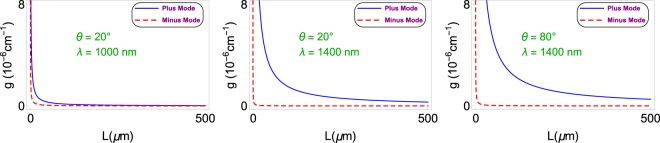
Gain coefficient as a function of TWS slab thickness *L* corresponding to various incidence angles and wavelengths. Increasing incidence angle and wavelength lead to higher gain values, and favor plus mode.

Thickness of the TWS slab plays a rather distinctive role for lasing. The thicker the TWS slab is, the higher quality factor is achieved. This is obviously seen in Fig. 6. We realize that the less gain is obtained once we choose a relatively small wavelength *λ* and incidence angle *θ*. As we increase *λ* and *θ*, required gain values of plus and minus modes increase, indeed differ considerably.

**Figure 7:**
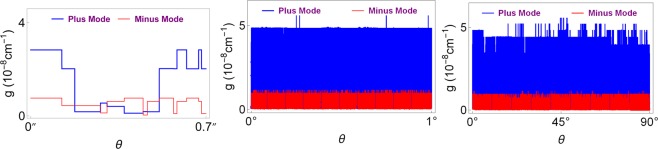
Gain coefficient as a function of incident angle *θ*. Here the symbol ″ simply refers to the Arcsecond, which is given by 1″ = 1°/3600. Notice that patterns are tightly packed once the range of the angle is increased.

**Figure 8:**
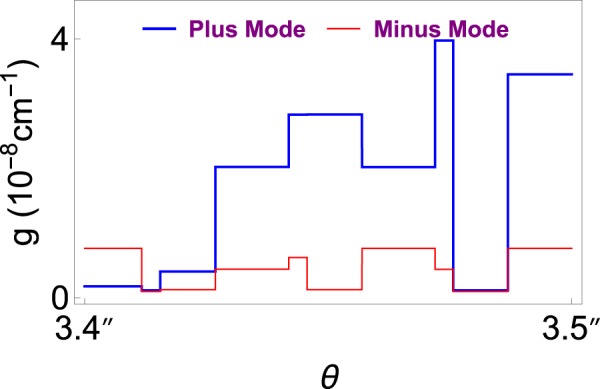
Schematic diagram of the gain *g* as a function of incident angle *θ* for the plus and minus lasing modes. Plus and minus modes overlap at angles *θ* ≈ 3.42″ and *θ* ≈ 3.46″ at the gain value *g* ≈ 1.17 × 10^−9^ cm^−1^.

Finally, Fig. 7 displays dependence of incidence angle on the gain coefficient *g* to achieve lasing. We immediately realize the quantized patterns of gain such that only certain values are allowed for the lasing threshold condition. This quantization behavior is a clear indication of topological feature of our Weyl semimetal system. Also, notice the compact patterns that occur in both plus and minus modes. Again minus mode arises at small gain values compared to the plus mode. One can easily obtain the bimodal lasing using these patterns by observing the overlapping positions of plus and minus modes. For instance, Fig. 8 shows one such a case for a TWS system with parameters of incident angles *θ* ≈ 3.42″ and *θ* ≈ 3.46″ at gain value *g* ≈ 1.17 × 10^−9^ cm^−1^.

## Effect of Dispersion

If there exists a dispersion in the refractive index ${\rm{n}}$, then we need to consider the effect of wavenumber *k* on ${\rm{n}}$. We imagine that active part of the TWS optical system with the gain ingredient is formed by doping a host medium of refractive index *n*_0_ and its refractive index satisfies the dispersion relation

30 $${{\mathfrak{n}}}^{2}={n}_{0}^{2}-\frac{{\hat{\omega }}_{p}^{2}}{{\hat{\omega }}^{2}-1+i\hat{\gamma }\hat{\omega }},$$

where $\hat{\omega }\,:\,=\omega /{\omega }_{0}$, $\hat{\gamma }\,:\,=\gamma /{\omega }_{0}$, ${\hat{\omega }}_{p}\,:\,={\omega }_{p}/{\omega }_{0}$, *ω*_0_ is the resonance frequency, *γ* is the damping coefficient, and *ω*_*p*_ is the plasma frequency. ${\hat{\omega }}_{p}^{2}$ can be described in the leading order of the imaginary part *κ*_0_ of ${\mathfrak{n}}$ at the resonance wavelength ${\lambda }_{0}\,:\,=2\pi c/{\omega }_{0}$ by the expression ${\hat{\omega }}_{p}^{2}=2{n}_{0}\hat{\gamma }{\kappa }_{0}$, where quadratic and higher order terms in *κ*_0_ are ignored^38^. By replacing this equation in (30), employing (5), and neglecting the quadratic and higher order terms in *κ*_0_, we obtain the real and imaginary parts of refractive index as follows^28,38^

31 $$\eta \approx {n}_{0}+\frac{{\kappa }_{0}\hat{\gamma }(1-{\hat{\omega }}^{2})}{{(1-{\hat{\omega }}^{2})}^{2}+{\hat{\gamma }}^{2}{\hat{\omega }}^{2}},\,\kappa \approx \frac{{\kappa }_{0}{\hat{\gamma }}^{2}\hat{\omega }}{{(1-{\hat{\omega }}^{2})}^{2}+{\hat{\gamma }}^{2}{\hat{\omega }}^{2}}$$

*κ*_0_ can be written as *κ*_0_ = −*λ*_0_*g*_0_/4*π* at resonance wavelength *λ*_0_. Substituting this relation in (31) and making use of (5) and (22), we can determine the *λ* and *g*_0_ values for the uni- or bimodal TWS laser. In our optical configuration, TWS slab material holds the following values of the parameters refractive index, resonance wavelength, and corresponding $\hat{\gamma }$ value^33,35–37^:

32 $$\begin{array}{c}{n}_{0}=6,\,{\lambda }_{0}=1378\,{\rm{nm}},\,\hat{\gamma }=0.033,\\ \theta ={30}^{\circ },\,L=1\,{\rm{cm}}.\end{array}$$

**Figure 9:**
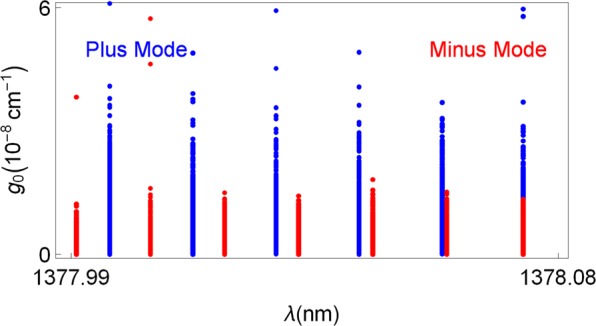
The threshold gain *g*_0_ as a function of wavelength *λ* corresponding to both plus and minus modes. Plus mode is represented by blue dots while minus modes by red ones. Overlapping dots which take place at wavelength *λ* ≈ 1378.0735 nm imply the bimodal positions.

Spectral singularity points which give rise to the lasing threshold conditions are explicitly calculated for various parameters in Table 2 for our setup of the TWS slab in (32). Figure 9 displays the positions of lasing points in *λ* − *g*_0_ plane around the resonance wavelength, which yield divergent quality factors. Obviously, the wavelength layout corresponding to each particular mode is incompatible with the reciprocal mode unless they do coincide, in which case a bimodal lasing occurs. In our case, *λ* ≈ 1378.0735 nm is the wavelength for the bimodal lasing. It is remarkable to realize that only particular discrete wavelengths allow lasing whereas threshold gain values are rather small compared to regular optical slab materials. This is encountered only with a TWS slab laser. If one considers different wavelength intervals, it is just enough to extend Fig. 9 to all wavelength ranges including ultraviolet, visible or infrared. But this time, corresponding gain values would be slightly higher since we move away from the resonance condition, see^28,39–41^.

**Table 2:** Types and corresponding configurations of a TWS slab laser with *L* = 1 cm and *θ* = 30°.

*κ*	*g*_0_ (cm^−1^)	*λ* (nm)	Type of Lasing
−1.800 × 10^−13^	1.6419 × 10^−8^	1377.997	Plus Mode
−6.341 × 10^−14^	5.7827 × 10^−9^	1378.278	Plus Mode
−1.539 × 10^−13^	1.4031 × 10^−8^	1378.0586	Plus Mode
−4.089 × 10^−14^	3.7286 × 10^−9^	1377.9909	Minus Mode
−1.238 × 10^−13^	1.1287 × 10^−8^	1378.0320	Minus Mode
−1.927 × 10^−14^	1.7572 × 10^−9^	1378.0594	Minus Mode
−1.237 × 10^−13^	1.1280 × 10^−8^	1378.0735	Bimodal
−6.554 × 10^−14^	5.9773 × 10^−9^	1378.0735	Bimodal

## Kerr and Faraday Rotations in Lasing and CPA

We now turn our attention to the discussion of the Kerr and Faraday rotations which need to be understood to unveil the lasing behaviors and characteristics inside and outside the TWS slab, and explicitly see their impressive features. Since lasing is structured by the spectral singularities, we impose the spectral singularity conditions given in (21) or (22), and then impose the conditions ${A}_{1}^{(-)}={A}_{2}^{(-)}={C}_{1}^{(+)}=0$ for plus-mode and ${A}_{1}^{(-)}={A}_{2}^{(-)}={C}_{2}^{(+)}=0$ for minus-mode lasing to obtain necessary configurations of electric fields inside and outside the TWS slab, see^29^ for details of this calculation. When we attain the spectrally singular fields, we can easily construct spectrally singular right and left circularly polarized fields *E*_±_(**z**), which are given by

$${E}_{+}({\bf{z}})={{\mathscr{F}}}_{+}({\bf{z}})\,{e}^{i{\mathfrak{K}}{\bf{x}}\tan \theta },\,{E}_{-}({\bf{z}})={{\mathscr{G}}}_{+}({\bf{z}})\,{e}^{i{\mathfrak{K}}{\bf{x}}\tan \theta }.$$

Hence, the Kerr and Faraday rotation angles are determined to be ${\theta }_{K}\,:\,=({\rm{\arg }}{E}_{+}({\bf{z}})-{\rm{\arg }}{E}_{-}({\bf{z}}))/2$ for **z** ∉ [0, 1] and ${\theta }_{F}\,:\,=({\rm{\arg }}{E}_{+}({\bf{z}})-{\rm{\arg }}{E}_{-}({\bf{z}}))/2$ for **z** ∈ [0, 1], respectively, where arg refers to the argument of corresponding quantity. Notice that when **z** < 0 of **z** > 1 we find the Kerr rotation angles *θ*_*K*_ which are equal in magnitude due to reciprocal response of the system. This observation leads to an important consequence about the CPA lasers built by TWS systems. The necessary and sufficient condition to generate a TWS CPA laser is to emit waves from both sides using polarizations exactly at Faraday angle *θ*_*F*_ with appropriate phases and magnitudes^29–31^. This is due to the main idea of a CPA that is time reversal symmetry of a regular laser^42–49^. Since this procedure requires special attention with excessive efforts, we reserve it for another study in its own right.

## Concluding Remarks

In this study, we reveal basics of constructing a TWS laser by means of the transfer matrix approach. We employ the known features of TWS such that presence of the Weyl nodes gives rise to Fermi arcs configurations on relevant surfaces leading to magneto-optical effects. Alignment of the Weyl nodes determines how these magneto-optical effects response to the scattering of TWS slab. If the electromagnetic waves fall on the surface which has Fermi arcs, Maxwell equations turn out to be in simple familiar form, and do not contain any topological term. Besides, interesting case occurs when the electromagnetic waves are exposed to the surface with no Fermi arcs, which is what we have explored in the present paper. This case induces Maxwell equations with topological terms which give rise to coupled Helmholtz equations. We observe that this coupling refers to the inherent feature of Kerr and Faraday rotations of a generic TWS slab.

Our discussion strongly embraces the transfer matrix method such that the boundary conditions are highly effective^28,32,50,51^. Since the basic characteristics of a TWS slab manifest itself by the effects of the surfaces, this method becomes notable in this respect. We found out the transfer matrix, and obtained the condition of spectral singularities which lead to construct a TWS slab laser. We notice that our one dimensional problem turns into a two dimensional one by virtue of the Kerr and Faraday rotations. This gives a bimodal laser system corresponding to effective refractive indices ${\tilde{{\mathfrak{n}}}}_{\pm }$ in (16), which generate lasing in plus and minus modes as discussed in (22). These distinctive modes are peculiar to a TWS laser system, which can also be observed in any material with magneto-optical effects. We find out that exact lasing modes of a TWS slab are provided by the spectral singularity condition in (21). In particular, the plus mode lasing provides bidirectional lasing due to ${\tilde{{\mathfrak{n}}}}_{+}$ and left-side lasing due to ${\tilde{{\mathfrak{n}}}}_{-}$, whereas the minus mode lasing yields bidirectional one due to ${\tilde{{\mathfrak{n}}}}_{-}$ and left-side lasing due to ${\tilde{{\mathfrak{n}}}}_{+}$, see Fig. 3.

Lasing characteristics of these modes are obtained by the optimal control of system parameters. We have seen that the required gain value for a minus mode lasing is always less that the one for a plus mode. This gain value must be quantized, which is due to topological feature of TWS system. Also, relatively smaller TWS slab thickness is sufficient for the minus mode lasing. It is revealed that incident angles corresponding to uni- or bimodal lasing is so sensitive to tiny variations, and depends on adjusting definite gain value. For some distinct angles with appropriate gain values, one can obtain bimodal lasing as seen in Fig. 8. This remarkable feature is explicitly seen in the case of dispersion effect in Fig. 9 such that only some specific wavelengths give rise bimodal lasing. Once all other parameters are fixed, uni- or bimodal lasing occurs only at discrete wavelengths. Moreover, increasing wavelength gives rise to higher Weyl node separations as in Fig. 4 when the remaining parameters are fixed. Considering all these behaviors of parameters, one can construct a highly effective TWS slab laser. We present some samples of these optimal parameters in Table 2.

As a final consequence, this work pioneers the construction of a topological CPA laser. For the realization of a TWS CPA, one needs to adjust appropriate configuration of the whole topological optical setup because all incoming waves has to be absorbed inside the slab. For this to occur, we replace the gain by the loss, employ exact phase and magnitudes of the incoming waves, and finally set the polarization angle to Faraday rotation angle which can be computed with the help of the spectral singularity condition. Because of the arrangement of the Faraday rotation angle, a TWS CPA could be challenging. Our findings figure out this problem. We expect the results of this paper to guide experimental attempts for the realization of a concrete TWS laser and CPA. As a future perspective, it could be intriguing to discuss the dynamical cases, wherein the high-intensity lasers may damage the properties of the Weyl points. It is noted that the dynamical Floquet theory may be more applicable than the static considerations in this case.

## Appendix

### Modified maxwell equations

In the low energy limit of a TWS, spatially varying axion term plays a significant role in determining its electromagnetic response. The full action of corresponding TWS slab system is described by the sum of conventional and axionic terms as *S* = *S*_0_ + *S*_Θ_

$$\begin{array}{rcl}{S}_{0} & = & \int \{-\frac{1}{4{\mu }_{0}}{F}_{\mu \nu }{F}^{\mu \nu }+\frac{1}{2}{F}_{\mu \nu }{{\mathscr{P}}}^{\mu \nu }-{J}^{\mu }{A}_{\mu }\}{d}^{3}xdt,\\ {S}_{\Theta } & = & \frac{\alpha }{8\pi {\mu }_{0}}\int \{\Theta (\overrightarrow{r},t){\varepsilon }^{\mu \nu \alpha \beta }{F}_{\mu \nu }{F}_{\alpha \beta }\}{d}^{3}x\,dt,\end{array}$$

where ${F}_{\mu \nu }={\partial }_{\mu }{A}_{\nu }-{\partial }_{\nu }{A}_{\mu }$, ${{\mathscr{P}}}^{\mu \nu }$ tensor represents the electric polarization and magnetization respectively by ${{\mathscr{P}}}^{0i}=c{P}^{i}$ and ${{\mathscr{P}}}^{ij}=-\,{\varepsilon }^{ijk}{M}_{k}$. *J*^*μ*^ is the electric current four-vector and *ε*^*μvαβ*^ is the totally antisymmetric tensor (*ε*^0123^ = 1). Space and time dependent axion term is given by $\Theta (\overrightarrow{r},t)=2\overrightarrow{b}\cdot \overrightarrow{r}-2{b}_{0}t$, where $\overrightarrow{b}$ and *b*_0_ denote the separation of nodes in momentum and energy space respectively. In our case, *b*_0_ is set to zero, since Weyl nodes are assumed to share the same chemical potential. If the action is varied with respect to *A*_*μ*_, the following equations of motion is obtained

33 $$-\frac{1}{{\mu }_{0}}{\partial }_{\nu }{F}^{\mu \nu }+{\partial }_{\nu }{{\mathscr{P}}}^{\mu \nu }+\frac{\alpha }{2\pi {\mu }_{0}}{\varepsilon }^{\mu \nu \alpha \beta }{\partial }_{\nu }(\Theta {F}_{\alpha \beta })={J}^{\mu }$$

It is obvious that expanding this equation yields the modified Maxwell equations given by Eqs. 1 and 2 in the presence of the axion field term. For more details of the derivation of Maxwell equations in a TWS material environment, see^52,53^.

### Computation of conductivities

To calculate the longitudinal *σ*_*yy*_ and transverse *σ*_*yx*_ conductivities of a TWS, we adopt the approaches given in^54–56^. We consider the simplest case with only two nodes located at $+\,\overrightarrow{b}$ and $-\,\overrightarrow{b}$, where $\overrightarrow{b}=b{\hat{e}}_{z}$. Near the Weyl nodes, linearized Hamiltonian is given by

$$H(\overrightarrow{k})=\pm \,\hslash {v}_{F}\overrightarrow{\sigma }\cdot (\overrightarrow{k}\pm \overrightarrow{b}).$$

where *v*_*F*_ is the Fermi velocity, and $\overrightarrow{\sigma }=({\sigma }_{x},{\sigma }_{y},{\sigma }_{z})$ is the vector whose components are Pauli matrices. Therefore, conductivity *σ*_*αβ*_ is obtained from Kubo formula as follows

$${\sigma }_{\alpha \beta }(\omega )=\frac{i}{\omega }\mathop{\mathrm{lim}}\limits_{q\to 0}{\Pi }_{\alpha \beta }(q,\omega )$$

In the absence of diamagnetic term, the polarization function ${\Pi }_{\alpha \beta }(q,\omega )$ is given by the current-current correlation function

$${\Pi }_{\alpha \beta }(q,i{\omega }_{n})=\frac{-1}{{\mathscr{V}}}{\int }_{0}^{\beta }\,d\tau \,{e}^{i{\omega }_{n}\tau }\langle {T}_{\tau }{\hat{{\mathscr{J}}}}_{\alpha }(q,\tau )|{\hat{{\mathscr{J}}}}_{\beta }(\,-\,q,0)\rangle .$$

where ${\mathscr{V}}$ is the volume of the system, and the current density operator $\hat{{\mathscr{J}}}$ is given by

$$\hat{{\mathscr{J}}}=-\,\frac{\delta H}{\delta \overrightarrow{A}}=\pm \,e{v}_{F}\overrightarrow{\sigma }.$$

Once we make analytic continuation $i{\omega }_{n}\to \omega +i{0}^{+}$, the real frequency behavior is obtained easily. Thus, for each node we obtain

$${\Pi }_{\alpha \beta }(\omega )=\frac{{e}^{2}{v}_{F}^{2}}{{\mathscr{V}}}\sum _{i,i{\prime} ,\overrightarrow{k}}\frac{f({\varepsilon }_{i{\prime} }(\overrightarrow{k}))-f({\varepsilon }_{i}(\overrightarrow{k}))}{\hslash \omega +{\varepsilon }_{i{\prime} }(\overrightarrow{k})-{\varepsilon }_{i}(\overrightarrow{k})+i{0}^{+}}\langle \overrightarrow{k}i|{\sigma }_{\alpha }|\overrightarrow{k}i{\prime} \rangle \langle \overrightarrow{k}i{\prime} |{\sigma }_{\beta }|\overrightarrow{k}i\rangle $$

where *f*(*x*) = 1/(1 + *e*^*βx*^) is the Fermi function. The expression $H(\overrightarrow{k})|\overrightarrow{k}i={\varepsilon }_{i}(\overrightarrow{k})|\overrightarrow{k}i$ with *i* = 1, 2 gives the quasiparticle energies and eigenstates. We can evaluate the longitudinal and transverse polarizations ${\Pi }_{\alpha \beta }(\omega )$ when the Fermi energy lies with nodes. Hence, in the low frequency limit, the longitudinal and transverse conductivities from both nodes are found to be expressions given in (7) and (8).

### Boundary conditions

Boundary conditions across the surface ${\mathscr{S}}$ between two regions of space are given by the statements: (1) Tangential component of electric field $\overrightarrow{E}$ is continuous across the interface, $\hat{n}\times ({\overrightarrow{E}}_{1}-{\overrightarrow{E}}_{2})=0$; (2) Normal component of magnetic field vector $\overrightarrow{B}$ is continuous, $\hat{n}\cdot ({\overrightarrow{B}}_{1}-{\overrightarrow{B}}_{2})=0$; (3) Normal component of electric flux density vector $\overrightarrow{D}$ is discontinuous by an amount equal to the surface current density, $\hat{n}\cdot ({\overrightarrow{D}}_{1}-{\overrightarrow{D}}_{2})={\rho }^{s}$; (4) Tangential component of the field $\overrightarrow{H}$ is discontinuous by an amount equal to the surface current density, $\hat{n}\times ({\overrightarrow{H}}_{1}-{\overrightarrow{H}}_{2})=\overrightarrow{{{\mathscr{J}}}^{s}}$. Here $\hat{n}$ represents the unit normal vector to the surface ${\mathscr{S}}$ from region 2 to region 1. In our optical configuration, we do not have the third condition since there is no normal component of the electric field. Thus, we obtain the boundary conditions as in Table 3, In this table, we used ${\mathfrak{K}}\,:\,={k}_{z}L$ and ${{\mathfrak{K}}}_{j}\,:\,={\mathfrak{K}}\,{\tilde{{\mathfrak{n}}}}_{j}$. *g*_*j*_ and *h*_*j*_ are defined as follows for convenience

34 $${g}_{j}\,:\,=\{\begin{array}{ll}+ & {\rm{for}}\,j=1,\\ - & {\rm{for}}\,j=2\end{array}\,{h}_{j}\,:\,=\{\begin{array}{ll}{\sigma }_{+} & {\rm{for}}\,j=1,\\ -{\sigma }_{-} & {\rm{for}}\,j=2\end{array}$$

where *σ*_±_ is specified by Eq. 20.

**Table 3:** Boundary conditions for TE waves corresponding to TWS slab. Here effective indices ${\tilde{{\rm{n}}}}_{\pm }$ are defined by (16), and functions *g*_*j*_ and *h*_*j*_ are given in (34).

**z** = 0	$$\begin{array}{l}\mathop{\sum }\limits_{j=1}^{2}[{A}_{j}^{(-)}+{C}_{j}^{(-)}]=\mathop{\sum }\limits_{j=-}^{+}[{B}_{1}^{(j)}+{B}_{2}^{(j)}],\\ \mathop{\sum }\limits_{j=1}^{2}{g}_{j}[{A}_{j}^{(-)}+{C}_{j}^{(-)}]=\mathop{\sum }\limits_{j=-}^{+}j[{B}_{1}^{(j)}+{B}_{2}^{(j)}],\\ \mu \mathop{\sum }\limits_{j=1}^{2}[({g}_{j}+2{h}_{j}){A}_{j}^{(-)}-({g}_{j}-2{h}_{j}){C}_{j}^{(-)}]=\mathop{\sum }\limits_{j=-}^{+}j{\tilde{{\mathfrak{n}}}}_{j}[{B}_{1}^{(j)}-{B}_{2}^{(j)}],\\ \mu \mathop{\sum }\limits_{j=1}^{2}[{A}_{j}^{(-)}-{C}_{j}^{(-)}]=\mathop{\sum }\limits_{j=-}^{+}{\tilde{{\mathfrak{n}}}}_{j}[{B}_{1}^{(j)}-{B}_{2}^{(j)}].\end{array}$$
**z** = 1	$$\begin{array}{l}\mathop{\sum }\limits_{j=1}^{2}[{A}_{j}^{(+)}{e}^{i{\rm{K}}}+{C}_{j}^{(+)}{e}^{-i{\mathfrak{K}}}]=\mathop{\sum }\limits_{j=-}^{+}[{B}_{1}^{(j)}{e}^{i{{\mathfrak{K}}}_{j}}+{B}_{2}^{(j)}{e}^{-i{{\mathfrak{K}}}_{j}}],\\ \mathop{\sum }\limits_{j=1}^{2}{g}_{j}[{A}_{j}^{(+)}{e}^{i{\mathfrak{K}}}+{C}_{j}^{(+)}{e}^{-i{\mathfrak{K}}}]=\mathop{\sum }\limits_{j=-}^{+}j[{B}_{1}^{(j)}{e}^{i{{\mathfrak{K}}}_{j}}+{B}_{2}^{(j)}{e}^{-i{{\mathfrak{K}}}_{j}}],\\ \mu \mathop{\sum }\limits_{j=1}^{2}[({g}_{j}+2{h}_{j}){A}_{j}^{(+)}{e}^{i{\mathfrak{K}}}-({g}_{j}-2{h}_{j}){C}_{j}^{(+)}{e}^{-i{\mathfrak{K}}}]=\mathop{\sum }\limits_{j=-}^{+}j{\tilde{{\mathfrak{n}}}}_{j}[{B}_{1}^{(j)}{e}^{i{{\mathfrak{K}}}_{j}}-{B}_{2}^{(j)}{e}^{-i{{\mathfrak{K}}}_{j}}],\\ \mu \mathop{\sum }\limits_{j=1}^{2}[{A}_{j}^{(+)}{e}^{i{\mathfrak{K}}}-{C}_{j}^{(+)}{e}^{-i{\mathfrak{K}}}]=\mathop{\sum }\limits_{j=-}^{+}{\tilde{{\mathfrak{n}}}}_{j}[{B}_{1}^{(j)}{e}^{i{{\mathfrak{K}}}_{j}}-{B}_{2}^{(j)}{e}^{-i{{\mathfrak{K}}}_{j}}].\end{array}$$
